# Association Between Postoperative Pancreatitis and Clinically Relevant Postoperative Pancreatic Fistula After Pancreaticoduodenectomy: A Prospective Observational Study From a Single Tertiary Care Center

**DOI:** 10.7759/cureus.102270

**Published:** 2026-01-25

**Authors:** Uday Sankar Reddy Kathulapalli, Harshal Bhoi, Jyotirmay Jena, Satyaprakash Ray Choudhury, Sumit Mohanty, Swamy Rajesh Hudugur

**Affiliations:** 1 Surgical Gastroenterology, Siksha 'O' Anusandhan, Institute of Medical Sciences and Sum Hospital, Bhubaneswar, IND

**Keywords:** clinically relevant popf, drain fluid amylase, pancreaticoduodenectomy, postoperative pancreatic fistula, risk stratification, roc analysis, serum amylase

## Abstract

Backgrounds/Aims: Postoperative pancreatic fistula remains a key determinant of morbidity following pancreaticoduodenectomy (PD). This study assessed whether postoperative day 1 (POD 1) serum amylase (SA) and drain fluid amylase (DFA) can predict clinically relevant postoperative pancreatic fistula (CR-POPF) to facilitate early risk-stratified postoperative management.

Methods: This prospective observational cohort study (January 2021-April 2023) included adult patients undergoing PD with duct-to-mucosa pancreaticojejunal anastomosis. SA and DFA levels were measured on POD 1, POD3, and POD 5. The primary outcome was CR-POPF, defined according to the 2016 International Study Group of Pancreatic Surgery criteria. Receiver operating characteristic (ROC) analyses were performed to identify optimal POD 1 cutoff values.

Results: Among 57 patients, nine (15.8%) developed CR-POPF. Mean POD 1 SA and DFA levels were significantly higher in patients who developed CR-POPF compared with those who did not (SA: 464 ± 164 vs 262 ± 176 IU/L, p = 0.002; DFA: 12,664 ± 8,800 vs 1,045 ± 1,128 IU/L, p < 0.001). POD 1 SA demonstrated an AUC of 0.803 (95% CI 0.677-0.897), with an optimal cutoff of 363 IU/L (sensitivity 77.78%, specificity 75.0%, negative predictive value (NPV) 94.7%). POD 1 DFA showed superior discrimination with an AUC of 0.889 (95% CI 0.778-0.957); a cutoff of 3,011 IU/L yielded a sensitivity of 66.67%, specificity of 97.92%, positive predictive value (PPV) of 85.7%, and NPV of 94.0%. A dilated pancreatic duct (>3 mm) was inversely associated with CR-POPF (p < 0.001).

Conclusions: POD 1 SA and DFA levels provide a reliable early prediction of CR-POPF following PD. A DFA threshold above 3,011 IU/L demonstrates high specificity for CR-POPF, whereas a SA level ≤363 IU/L effectively excludes its occurrence. Incorporation of these parameters into postoperative protocols may aid early drain management decisions and targeted intervention in high-risk patients.

## Introduction

Pancreaticoduodenectomy (PD), or the Whipple procedure, is a complex surgical intervention involving the resection of the pancreatic head, uncinate process, duodenum, proximal jejunum, distal bile duct, gallbladder, and often part of the stomach, primarily performed to treat malignancies in the pancreatic head, periampullary region, and distal bile duct, as well as certain benign or premalignant conditions, with bilioenteric continuity restored post-resection [1]. Despite significant advancements over the decades, pancreaticoduodenectomy remains associated with high morbidity rates, reaching up to 60%, and mortality rates ranging from 2% to 10% [1]. Postoperative complications following pancreaticoduodenectomy include delayed gastric emptying, pancreatic fistulas, visceral artery pseudoaneurysms, bile leaks, and exocrine and endocrine insufficiency [2-6].

Among these, postoperative pancreatic fistulas are a significant concern, defined by amylase levels exceeding three times the upper normal serum limit in the pancreatic drain on or after postoperative day 3 (POD 3) [7]. They are classified into three grades: Grade A as biochemical leaks with no clinical relevance, Grade B as persistent amylase-rich drainage requiring prolonged management, and Grade C as fistulas causing organ failure [8]. The pathophysiology of postoperative pancreatic fistula (POPF) is poorly understood and often misattributed to mechanical anastomotic failure, despite varied surgical methods like fibrin sealants, tissue patches, and stents showing limited efficacy in reducing POPF rates, while risk factors including soft gland texture, small duct diameter, non-cancer pathology, and high blood loss predict POPF using the Fistula Risk Score (FRS), with less-validated factors like BMI, fluid administration, and nutrition being understudied, and advanced age linked to higher perioperative mortality but not increased POPF risk [9-12]. Early predictive methods for POPF after pancreatic resection include biochemical markers such as serum amylase (SA), lipase, and urinary trypsinogen-2 measured on the first postoperative day (POD 1) and histological evaluation of the pancreatic neck margin for acinar cell density showing predictive value, which can be assessed intraoperatively during frozen sections [13]. These methods may provide more precise and quantitative characterization of the remnant gland than traditional assessments of glandular texture. Numerous studies highlight the utility of early drain fluid amylase (DFA) levels, particularly DFA1, in predicting postoperative pancreatic fistula (PF), with thresholds ranging from 100 to 5,000 U/L, but lack consensus on an optimal range for universal diagnosis, emphasizing the need for further research to standardize DFA thresholds and integrate them with clinical indicators for improved postoperative management [14-16].

The aim of this study was to evaluate the utility of POD 1 SA and DFA levels. The objectives were to determine the most effective thresholds for POD-1 SA and DFA levels for predicting clinically relevant postoperative pancreatic fistula (CR-POPF) and to provide insights into optimizing postoperative management based on these predictive factors.

## Materials and methods

This prospective observational cohort study was conducted at the Department of Surgical Gastroenterology, Institute of Medical Sciences and SUM Hospital, Bhubaneswar, Odisha, from January 2021 to April 2023, after obtaining ethical approval from the Institutional Ethical Committee. The study population included patients aged 18 years or older who underwent PD with a pancreaticojejunal duct-to-mucosa anastomosis. Patients under 18 years, those unwilling to provide informed consent, or those who died within the first 48 hours post-surgery for reasons unrelated to the study were excluded. The intervention involved analyzing SA and DFA levels on POD 1, 3, and 5 (wherever applicable), while the comparator was the absence of CR-POPF [17-18]. The primary outcome was the identification and grading of CR-POPF based on the 2016 criteria by the International Study Group of Pancreatic Fistula (ISGPF), along with categorization of other complications per International Study Group of Pancreatic Surgery (ISGPS) definitions [19-20]. The study setting included the collection of clinical and demographic data using a predefined proforma, with patients categorized into CR-POPF and no CR-POPF groups for comparative analysis to correlate amylase levels with clinical outcomes.

The collected data were systematically recorded into a Microsoft Excel spreadsheet (Microsoft Corp., Redmond, WA) and subjected to comprehensive statistical analysis using SPSS version 22 software (IBM Corp., Armonk, NY) to ensure methodological rigor [21]. Categorical variables were summarized as frequencies and proportions, with their associations tested using chi-square or Fisher's exact test to account for small sample sizes and ensure precision in categorical comparisons [22]. Continuous variables were presented as mean and standard deviation, with their distribution assessed using both the Kolmogorov-Smirnov and Shapiro-Wilk tests to validate normality assumptions [23].

For normally distributed data, the independent t-test was employed to evaluate the significance of differences in mean values, providing robust insight into group-level variations. Univariate analysis was meticulously performed to explore the relationship between multiple risk factors and CR-POPF, enabling identification of key predictors.

Receiver operating characteristic (ROC) curve analysis was conducted to determine the optimal thresholds for SA and DFA levels, based on their sensitivity and specificity. This approach ensured that the cut-off points were not only statistically robust but clinically applicable, enhancing the study's predictive capacity. Collectively, this multi-layered analytical framework underscores the robustness and reliability of the methodology adopted for this study.

## Results

**Figure 1:**
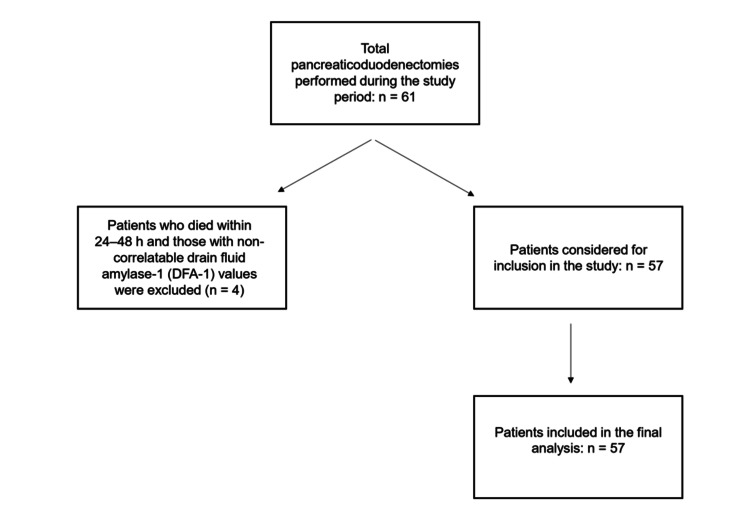
CONSORT diagram CONSORT: Consolidated Standards of Reporting Trials

Initially, 61 patients were enrolled in the study, out of which four had in-hospital mortality due to cardiac causes and were excluded from the study. A total of 57 patients completed the study and were included for the final analysis. All patients underwent pancreaticoduodenectomy with pancreaticojejunal anastomosis by duct to mucosa technique, as mentioned in Figure 1.

The demographic details of the participants are provided in Table 1. The mean age of the study population was 54.04 ± 11.83 years, with the majority (33.3%) aged between 46 and 55 years. The most common presenting symptom was abdominal pain (80.7%), followed by jaundice (52.6%), vomiting (22.8%), fever (21.1%), and anorexia (19.3%). Cholangitis was observed in 26.3% of patients. Preoperative biliary stenting was noted in 54.4% of cases, although the reasons for stenting were often unclear due to patient referrals. The pancreas exhibited a soft consistency in 84.2% of patients and a firm texture in 15.8%. Dilation of the main pancreatic duct (≥3 mm) was present in 59.6%, while the common bile duct (≥8 mm) was dilated in 82.5%. Mean intraoperative blood loss was 348 ± 120.23 mL. Histopathological analysis revealed malignant pathology in 91.2% of patients and benign pathology in 8.8%, reflecting the predominance of oncological indications for surgery.

**Table 1:** Demographic and clinical characteristics of the patients in study population The data is presented in numbers (percentages); means and standard deviations were used wherever indicated. SD: standard deviation; CCI: Charlson Comorbidity Index; MPD: main pancreatic duct; CBD: common bile duct; ICU: intensive care unit; POPF: postoperative pancreatic fistula; CR-POPF: clinically relevant postoperative pancreatic fistula; PPH: postpancreatectomy hemorrhage; DGE: delayed gastric emptying

Variables	Number of patients (%); N=57
Age (Mean + SD)	54.04 + 11.83
Sex (Male : Female)	1.47:1
Presenting symptoms	
Abdominal pain	46 (80.7%)
Jaundice	30 (52.6%)
Vomiting	13 (22.8%)
Fever	12 (21.1%)
Loss of appetite	11 (19.3%)
Melena	3 (5.3%)
Pruritus	3 (5.3%)
Weakness	3 (5.3%)
CCI [24]	
0	47 (82.5%)
1	8 (14.0%)
2	2 (3.5%)
Total bilirubin (mg%)	3.54
Intraoperative findings	
Texture: soft	48 (84.2%)
Texture: firm	9 (15.8%)
MPD: Dilated	34 (59.6%)
CBD: Dilated	47 (82.5%)
Cholangitis	15(26.31%)
Biliary stenting	31(54.4%)
Histology	
Benign	5(8.8%)
Malignant	52(91.2%)
Blood loss in ml (mean + SD)	348 + 120.23
ICU Stay in days (mean + SD)	2.12 + 1.32
Complication	45 (78.9%)
POPF	42 (73.7%)
Biochemical Leak	33 (57.9%)
CR-POPF	9 (15.8%)
PPH [25]	3 (5.3%)
Grade A	1 (1.8%)
Grade B	1 (1.8%)
Grade C	1 (1.8%)
DGE [26]	16 (28.1%)
Grade A	13 (22.8%)
Grade B	3 (5.3%)
Surgical site infection	6 (10.5%)
Bile leak	1 (1.8%)

Grade A POPF, also known as biochemical leak, was present in 33 (57.9%) patients. Grade B POPF was present in eight (14%) and Grade C POPF was present in one (1.8%) patient. In total, nine (15.8%) patients had CR-POPF in our study (Table 2). All nine patients were started on octreotide injection; two of these patients needed percutaneous drainage because of an intra-abdominal collection, and one patient required re-exploration. Delayed gastric emptying (DGE) was present in 16 (28.1%) patients, and postpancreatectomy hemorrhage was present in 3 (5.3%) patients. All the patients with DGE were managed conservatively with prokinetics. Two out of three patients with PPH required angioembolisation. The abdominal drains were removed on POD3, when there was no POPF; and in all others, they were kept further depending on the clinical parameters.

**Table 2:** Distribution of CR-POPF CR-POPF: clinically relevant postoperative pancreatic fistula

Parameters		Count	%
CR-POPF	No	48	84.2%
	Yes	9	15.8%
	Total	57	100.0%

The study population was categorized into two groups: patients with clinically relevant postoperative pancreatic fistula (CR-POPF) and those without CR-POPF, based on the updated International Study Group of Pancreatic Fistula (ISGPF) 2016 criteria. Comparative analysis was performed to identify differences in clinical and demographic parameters between these groups. Significant associations were observed with key parameters, highlighting potential predictors of CR-POPF. Table 3 outlines the baseline and intraoperative characteristics of patients in the CR-POPF and no CR-POPF groups. Notably, all patients in the CR-POPF group had a soft pancreatic gland texture, compared to 81.3% in the no CR-POPF group, though this difference was not statistically significant (P=0.157). However, pancreatic duct dilation (>3 mm) was significantly less frequent in the CR-POPF group (11.1%) compared to the no CR-POPF group (68.8%) (P<0.001). Preoperative and intraoperative factors such as blood loss, bile duct dilation, and site of pathology showed no significant differences between the groups.

**Table 3:** Comparison of risk factors between patients with and without CR-POPF CR-POPF: clinically relevant postoperative pancreatic fistula; CCI: Charlson Comorbidity Index; ND: not dilated; CBD: common bile duct; DCCA: distal cholangiocarcinoma; HOP: head of pancreas

Variables	CR-POPF [27]	No CR-POPF	Test Statistic (t/χ²)	Statistical Test	P-value
Age	48.2±9.1	55.1±12	t = -1.63	Independent t-test	0.109
Gender			χ² = 0.07	Chi-square test	0.78
Male	5 (55.6)	29 (60.4)			
Female	4 (44.4)	19 (39.6)			
CCI [24]			χ² = 0.494	Chi-square test	0.781
0	8 (88.9)	39 (81.3)			
1	1 (11.1)	7 (14.6)			
2	0 (0)	2 (4.2)			
Gland texture			χ² = 2.0	Chi-square test (Fisher's exact test)	0.157
Soft	9 (100)	39 (81.3)			
Firm	0 (0)	9 (18.7)			
Pancreatic duct			χ² = 10.5	Chi-square test	<0.001
Dilated	1 (11.1)	33 (68.8)			
Not dilated	8 (88.9)	18 (31.3)			
CBD [28]			χ² = 2.23	Chi-square test (Fisher's exact test)	0.525
Dilated	7	40			
Not dilated	2	8			
Site (primary site of pathology) [29]			χ² = 8.94	Chi-square test	0.063
Periampullary	5 (55.6)	36 (75)			
Benign	1 (11.1)	4 (8.3)			
DCCA ()	3 (33.3)	2 (4.2)			
HOP [30]	0 (0)	1 (2.1)			
Duodenum	0 (0)	5 (10.4)			
Pre-operative bilirubin total	2.51±0.5	3.74±1.5	t = -0.607	Independent t-test	0.546
Pre-operative bilirubin direct	2.04±0.3	2.93±1.02	t = -0.522	Independent t-test	0.603
Albumin	3.73±0.74	3.74±0.57	t = -0.03	Independent t-test	0.971
Hemoglobin	11.38±2.3	10.52±1.79	t = 1.249	Independent t-test	0.217
Blood loss	369 ±130	345±119	t = 0.539	Independent t-test	0.592
Day 1					
Serum	464±164	262±176	t = 3.19	Independent t-test	0.002
Drain	12664±8800	1045±1128	t = 5.63	Independent t-test	<0.001
Day 3					
Serum	197±120	89.6±68.2	t = 3.78	Independent t-test	<0.001
Drain	5072±6676	1265.8±534	t = 2.96	Independent t-test	0.005
Day 5					
Serum	211±325	58.5±41.2	t = 3.24	Independent t-test	0.002
Drain	7356±534	931.5±261	t = 2.68	Independent t-test	0.010

Table 4 highlights the results of univariate analysis, which identified dilated pancreatic duct (>3 mm), SA levels, and DFA levels on POD1 as significant predictors of CR-POPF (P<0.05). The mean POD1 SA level was significantly higher in the CR-POPF group (464 ± 164 IU/L) compared to the no CR-POPF group (262 ± 176 IU/L) (P=0.002). Similarly, the mean POD1 DFA level was markedly elevated in the CR-POPF group (12,664 ± 8,800 IU/L) versus the no CR-POPF group (1,045 ± 1,128 IU/L) (P<0.001). These findings suggest a strong correlation between early postoperative amylase levels and the development of CR-POPF.

**Table 4:** Results of univariate analysis for risk factors associated with CR-POPF CR-POPF: clinically relevant postoperative pancreatic fistula; CBD: common bile duct; HPE: histopathological examination; DFA: drain fluid amylase; SA: serum amylase

Factor	Estimate (β)	SE	OR	Statistical Test	p-value
Age in years (<50yrs - >50yrs)	1.29	0.769	3.65	Univariate logistic regression	0.092
Sex (male-female)	0.2	0.733	1.22	Univariate logistic regression	0.785
CCI index [24]					
1-0	0.362	1.137	1.44	Univariate logistic regression	0.75
2-0	14.982	1696.734	0.000003	Univariate logistic regression	0.993
Preoperative albumin <3/>3	1.145	0.957	3.14	Univariate logistic regression	0.231
Preoperative CBD stenting (yes/no)	1.676	0.854	5.34	Univariate logistic regression	0.05
Blood loss <500ml/>500ml	0.318	1.182	0.727	Univariate logistic regression	0.788
Gland texture (firm/soft)	17.1	2174	0.0002	Univariate logistic regression	0.994
Pancreatic duct (<3mm/>3mm)	2.868	1.105	17.6	Univariate logistic regression	0.009
HPE (malignancy/benign)	0.134	1.15	0.875	Univariate logistic regression	0.907
Day 1 SA	0.006	0.00227	0.994	Univariate logistic regression	0.008
Day 1 DFA	4.39	1.079	4.07	Univariate logistic regression	<0.001
Day 3 SA	0.0126	0.00442	0.988	Univariate logistic regression	0.004
Day 3 DFA	0.0015	0.00053	0.998	Univariate logistic regression	0.005
Day 5 SA	0.0115	0.00569	0.989	Univariate logistic regression	0.043
Day 5 DFA	0	0.000071	0.998	Univariate logistic regression	0.024

Table 5 presents the results of the ROC analysis for POD1 SA in predicting clinically relevant postoperative pancreatic fistula (CR-POPF). The area under the ROC curve (AUC) for POD1 SA was 0.803, with a 95% confidence interval of 0.667-0.897, indicating good discriminatory power (P<0.001). The optimal cutoff for SA on POD1 was determined to be 363 IU/L, yielding a sensitivity of 77.78% and a specificity of 75%. The positive predictive value (PPV) was 36.8%, and the negative predictive value (NPV) was remarkably high at 94.7%, emphasizing the reliability of this threshold for ruling out CR-POPF in low-risk patients.

**Table 5:** Postoperative day 1 serum amylase as a predictor of clinically relevant postoperative pancreatic fistula ROC: receiver operating characteristic; AUC: area under the curve

Area under the ROC curve (AUC)	0.803
Standard error	0.0681
95% Confidence interval	0.677 to 0.897
Z statistic	4.452
Significance level P (area=0.5)	<0.0001
Youden index J	0.5278
Associated criterion	>363

**Figure 2:**
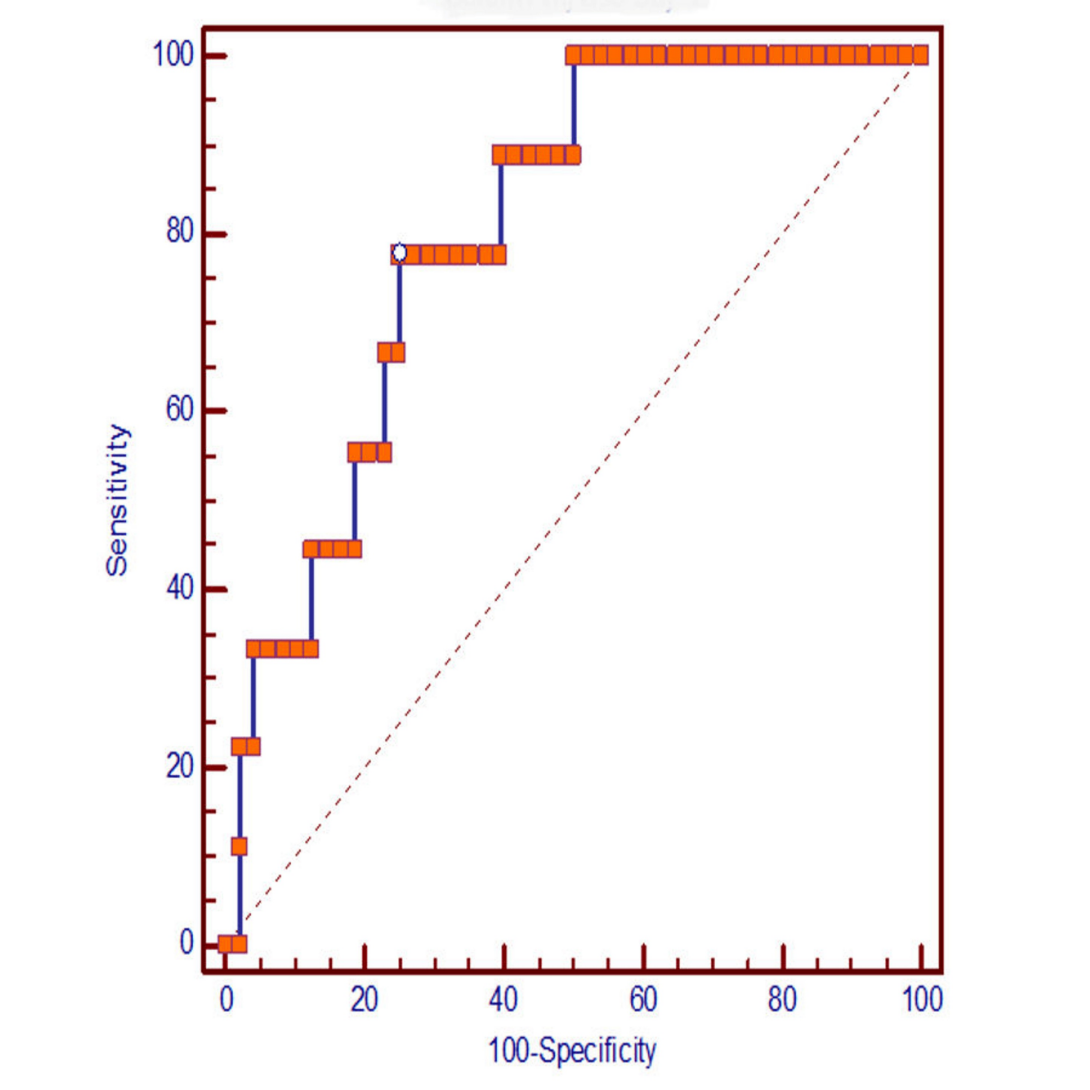
ROC curve analysis of postoperative day 1 serum amylase in predicting clinically relevant postoperative pancreatic fistula

Figure 2 depicts the ROC curve for POD1 SA levels, visually representing the trade-off between sensitivity and specificity at various thresholds. The curve highlights the strong predictive capability of SA, with an AUC of 0.803. The point corresponding to the cutoff value of 363 IU/L is marked, demonstrating its optimal balance between sensitivity (77.78%) and specificity (75%).

Table 6 details the ROC analysis for POD1 DFA levels in predicting CR-POPF. The AUC was 0.889, with a 95% confidence interval of 0.778-0.957, demonstrating excellent discriminatory power (P<0.0001). The optimal cutoff level for DFA on POD1 was identified as 3011 IU/L, achieving a sensitivity of 66.67% and an impressive specificity of 97.92%. The PPV was 85.7%, indicating a high likelihood of CR-POPF when DFA exceeded this threshold, while the NPV was 94.0%, confirming the cutoff’s utility in excluding CR-POPF in low-risk cases.

**Table 6:** Drain (PJ) Amylase Day 1 in prediction of CR-POPF CR-POPF: clinically relevant postoperative pancreatic fistula

Area under the ROC curve (AUC)	0.889
Standard Error	0.0633
95% Confidence interval	0.778 to 0.957
Z statistic	6.140
Significance level P (Area=0.5)	<0.0001

**Figure 3:**
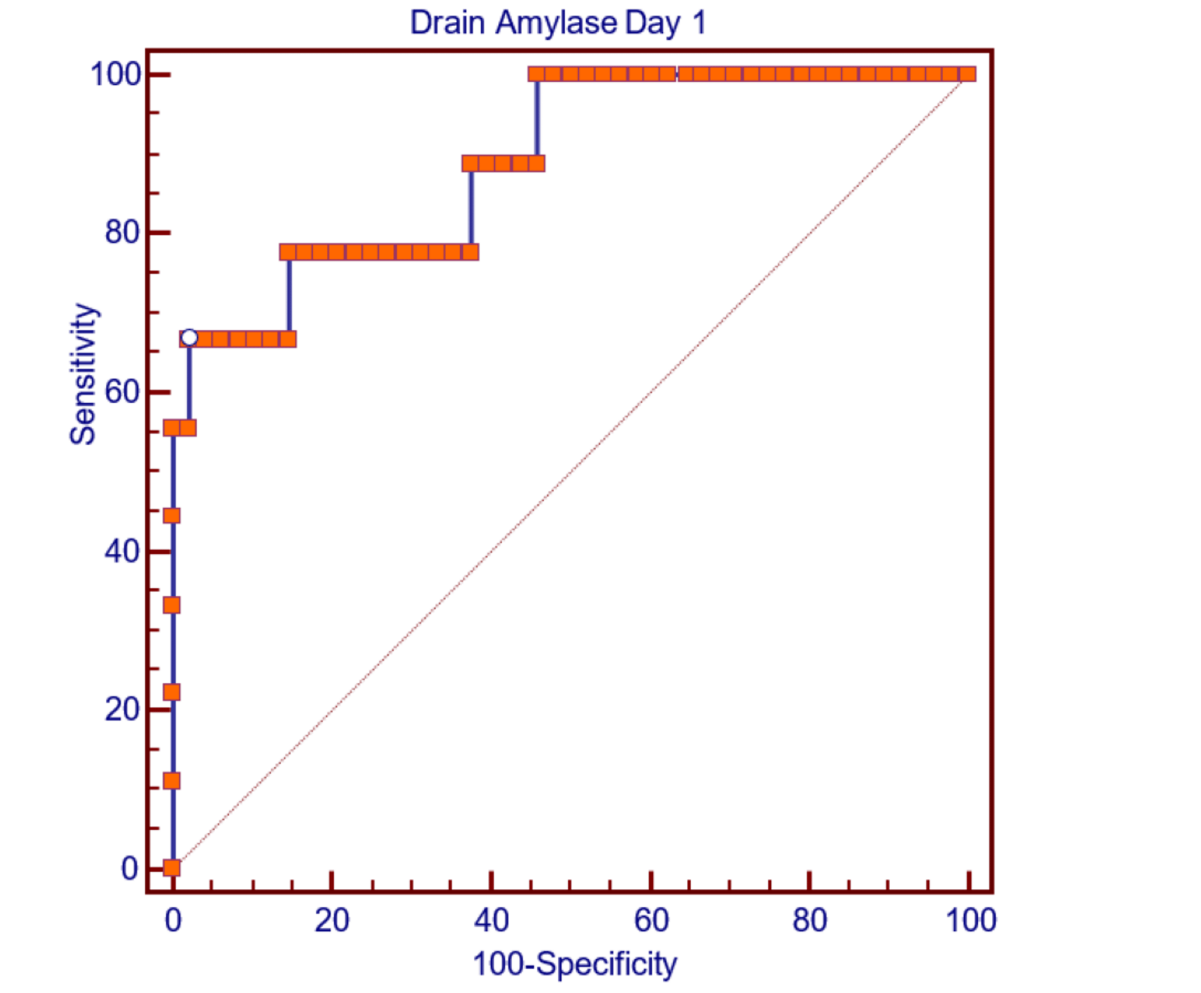
ROC curve showing postoperative day 1 pancreaticojejunostomy drain amylase for the prediction of CR-POPF ROC: Receiver operating characteristic; CR-POPF: clinically relevant postoperative pancreatic fistula

Figure 3 illustrates the ROC curve for POD1 DFA levels, showcasing an even higher predictive accuracy with an AUC of 0.889. The curve indicates excellent discriminatory power, with the cutoff value of 3011 IU/L marked, emphasizing its specificity (97.92%) and high PPV (85.7%). This figure underscores the robustness of DFA as a predictive marker for CR-POPF.

## Discussion

Pancreaticoduodenectomy is still considered to be a highly morbid procedure with a morbidity rate ranging from ranging from 20% to 60%. POPF has been one of the major contributors to postoperative morbidities, and early detection of the POPF and early intervention might improve the outcomes [31].

Herein, we presented the data of 57 consecutive patients who underwent PD in the Department of Surgical Gastroenterology for various causes, which include benign and malignant causes. We had "no POPF" in 26.3% of the patients. Grade A POPF, which is now termed "biochemical leak," was present in 33 patients out of 57 patients, accounting for 57.9%. Grade B POPF was present in eight of 57 patients, accounting for 14%, and Grade C POPF was present in one patient, accounting for 1.8% of total patients. In total, nine (15.8%) patients had CR-POPF in our study.

There are conflicting results regarding the placement of abdominal drains after pancreaticoduodenectomy [32,33]. We routinely put two drains in the abdomen; one in the sub-hepatic space, near hepaticojejunostomy anastomosis, and another near pancreaticojejunostomy site through the left flank. Enhanced recovery after surgery (ERAS) pathways are now widely applied to all abdominal surgeries, including pancreaticoduodenectomy, which emphasize early drain removal within 72 hours in low-risk patients [34]. Bassi et al concluded that early removal of drains is associated with a low risk of pancreatic fistula, intra-abdominal and pulmonary complications, hospital stay, and cost in their randomised control trial [35].

Several studies have explored the utility of DFA levels in predicting CR-POPF after PD, providing valuable insights for comparison. Molinari et al. [36] used a DFA1 cut-off of 5000 IU/L in their study, achieving a sensitivity of 93% and specificity of 84%. The POPF rate in their study was 19.7%. Kawai et al. [37] employed a DFA1 cut-off of 4000 IU/L and reported a sensitivity of 62% and specificity of 89%. The POPF rate in their study was 30.2%. Jin et al. [38] identified a cut-off amylase level of 2365.5 U/L in drain fluid on POD1, which predicted POPF with 78.6% sensitivity, 80% specificity, 66.7% PPV, and 88% NPV. Sutcliffe et al. [17] investigated a DFA1 cut-off of 350 IU/L and reported a sensitivity of 100% and specificity of 79%. The POPF rate in their study was 12.9%. Israel et al. [16] used a cut-off level of 100 IU/L and reported a sensitivity of 96% and specificity of 69% [16]. These studies suggest that very low DFA1 levels may have a higher sensitivity in detecting POPF, but at the cost of decreased specificity.

When compared to these similar studies, as shown in Table 7, our results demonstrate a relatively high specificity, indicating a low false-positive rate, which is important for the accurate prediction of POPF. The sensitivity of our study is within a comparable range, indicating a reasonable ability to identify patients at risk of developing POPF. Additionally, we predicted the incidence of CR-POPF. POD-1 SA, at a level > 363 IU/L, had a sensitivity of 77.78%, specificity of 75%, PPV of 36.8%, and a high NPV of 94.7% in predicting CR-POPF. POD-1 DFA, at a cut-off level of >3011 predicted CR-POPF with a sensitivity of 66.67%, specificity of 97.92%, PPV of 85.7%, and NPV 0f 94%. The high specificity (97.92%) and NPV (94.0%) observed for DFA on POD-1 in our study indicate that a drain amylase level above the identified threshold is highly indicative of CR-POPF, and patients with amylase levels below the threshold are less likely to develop this complication. However, it comes with moderate sensitivity (66.67%) and PPV (85.7%).

**Table 7:** Comparison of our study to other similar studies

Study	DFA1 cutoff IU/L	No of patients	PF rate	Sensitivity%	Specificity%	PPV%	NPV%	AUC	P-value
Molinari et al. [36]	5000	137	19.7	93	84	59	98	0.922	<0.001
Kawai et al. [37]	4000	1239	30.2	62	89	85	51	0.84	<0.001
Jin et al. [38]	2365	83	24	78	80	67	88	0.844	0.009
Sutcliffe et al. [17]	350	70	12.9	100	79	41	100	0.962	<0.0001
Israel et al. [16]	100	63	43	96	69	71	96	0.903	<0.001
Our study	3011	57	15.8	67	97.92	85.7	94	0.889	

The recent concept of etiopathogenesis of POPF shows its relationship to acinar cell density and postoperative pancreatitis, which in turn is reflected as high intraoperative amylase concentration (IOAC), high SA/lipase concentration, or urinary trypsinogen (UT) concentration [16,17]. High level of SA concentration on POD1 may indicate the ongoing pancreatitis in the remnant pancreas, maybe inflammatory or ischemic [18]. Similarly, the DFA levels on POD1 may reflect the mechanical disruption of pancreaticojejunostomy or, indirectly, the IOAC. The mechanical disruption is highly unlikely in the first 24 hours of surgery when it is performed, adhering to the principle of tension-free, well-vascularised anastomosis. Hence, POD1 DFA is an indirect measure of the IOAC, which has been proposed as an alternative pathogenesis of POPF after pancreatic resections. Our study adds evidence towards the postoperative pancreatitis as an etiopathogenesis of POPF. However, a large-scale study comparing all parameters is essential to further validate the hypothesis.

Limitations

This study was conducted at a single tertiary care center with a relatively small cohort of 57 patients, which may limit the generalizability of the findings and reduce the statistical power of the ROC-derived cutoff values. Due to the modest sample size, a multivariate analysis could not be performed, preventing adjustment for potential confounding factors. Furthermore, the inclusion of both benign and malignant pathologies, as well as variability in pancreatic texture and duct size, may have introduced biological heterogeneity influencing postoperative amylase dynamics. The DFA levels could also have been affected by differences in drain placement, output, and sampling technique despite standardized postoperative protocols. Intraoperative biochemical and histological parameters, such as intraoperative amylase concentration and acinar cell density, were not assessed, which might have provided deeper insight into the pathophysiology of POPF. Lastly, as an observational study without external validation, the amylase cutoff thresholds identified should be interpreted cautiously and confirmed in larger multicenter cohorts before being adopted into clinical practice.

## Conclusions

This study provides significant evidence that POD 1 DFA and SA levels are powerful and reliable predictors of CR-POPF following pylorus-resecting pancreaticoduodenectomy with pancreaticojejunal duct-to-mucosa anastomosis. By establishing precise thresholds, DFA >3011 IU/L and SA >363 IU/L, with exceptional specificity (97.92%) and negative predictive value (94%), this research not only advances the predictive accuracy for CR-POPF but also challenges traditional paradigms of postoperative management. These findings redefine early risk stratification, emphasizing the potential for safer, earlier drain removal and reduced morbidity, thereby setting a transformative benchmark in pancreatic surgery.
